# Chondrocyte autophagy mechanism and therapeutic prospects in osteoarthritis

**DOI:** 10.3389/fcell.2024.1472613

**Published:** 2024-10-23

**Authors:** Lan Li, Jie Li, Jian-Jiang Li, Huan Zhou, Xing-Wang Zhu, Ping-Heng Zhang, Bo Huang, Wen-Ting Zhao, Xiao-Feng Zhao, En-Sheng Chen

**Affiliations:** ^1^ Southern Medical University Hospital of Integrated Traditional Chinese and Western Medicine, Southern Medical University, Guangzhou, Guangdong, China; ^2^ School of Traditional Chinese Medicine, Southern Medical University, Guangzhou, Guangdong, China; ^3^ Affiliated Foshan Maternity and Child Healthcare Hospital, Southern Medical University, Foshan, Guangdong, China

**Keywords:** autophagy, chondrocytes, osteoarthritis, apoptosis, senescence

## Abstract

Osteoarthritis (OA) is the most common type of arthritis characterized by progressive cartilage degradation, with its pathogenesis closely related to chondrocyte autophagy. Chondrocytes are the only cells in articular cartilage, and the function of chondrocytes plays a vital role in maintaining articular cartilage homeostasis. Autophagy, an intracellular degradation system that regulates energy metabolism in cells, plays an incredibly important role in OA. During the early stages of OA, autophagy is enhanced in chondrocytes, acting as an adaptive mechanism to protect them from various environmental changes. However, with the progress of OA, chondrocyte autophagy gradually decreases, leading to the accumulation of damaged organelles and macromolecules within the cell, prompting chondrocyte apoptosis. Numerous studies have shown that cartilage degradation is influenced by the senescence and apoptosis of chondrocytes, which are associated with reduced autophagy. The relationship between autophagy, senescence, and apoptosis is complex. While autophagy is generally believed to inhibit cellular senescence and apoptosis to promote cell survival, recent studies have shown that some proteins are degraded by selective autophagy, leading to the secretion of the senescence-associated secretory phenotype (SASP) or increased SA-β-Gal activity in senescent cells within the damaged region of human OA cartilage. Autophagy activation may lead to different outcomes depending on the timing, duration, or type of its activation. Thus, our study explored the complex relationship between chondrocyte autophagy and OA, as well as the related regulatory molecules and signaling pathways, providing new insights for the future development of safe and effective drugs targeting chondrocyte autophagy to improve OA.

## 1 Introduction

OA is the most common type of arthritis. The prevalence of OA increases significantly with age, and the mechanisms leading to joint damage may be including oxidative damage, thinning of cartilage, muscle weakening, and a reduction in proprioception. OA is relatively rare in individuals under the age of 30, but among those aged over 75, approximately one-third experience symptomatic knee OA (Katz et al., 2021). Due to its high prevalence and the substantial disability it causes, OA is the leading cause of disability among the elderly. Joint vulnerability and joint load are two major factors that trigger OA. Joint vulnerability is associated with age, sex, genetics, abnormal changes in joint anatomy, and injury. Joint load is mainly associated with obesity and repeated use of joints (Palazzo et al., 2016). These two groups of risk factors interact in complex ways to contribute to the development of OA. Recent reports indicate that OA ranks as the second leading cause of burden in musculoskeletal diseases, accounting for about 7.1% of the total burden, and its burden increased significantly by 63.1% between 1990 and 2007.

**FIGURE 1:**
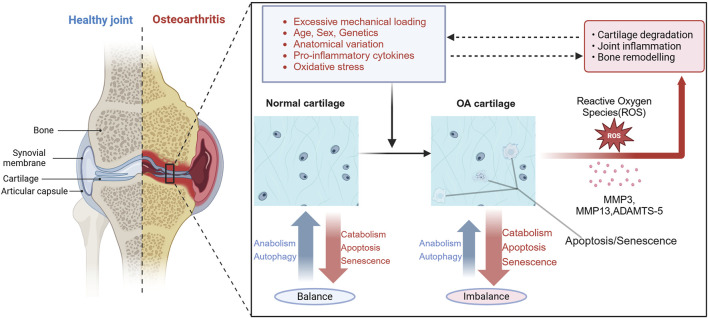
Overview of the pathogenesis of OA. Current research indicates that osteoarthritis is mainly associated with excessive mechanical loading, age, gender, genetics, anatomical variations, pro-inflammatory cytokines, and oxidative stress. These factors can lead to decreased chondrocyte autophagy, increased senescence and apoptosis, enhanced generation of reactive oxygen species (ROS), and expression of matrix-degrading enzymes, such as ADAMTS-4, ADAMTS-5, MMP3 and MMP13. This imbalance disrupts the balance between anabolism and catabolism in cartilage, leading to cartilage degeneration, joint inflammation, and bone remodeling, which further impedes the normal transmission of mechanical loading to chondrocytes. Dysfunctional chondrocytes contribute to impaired maintenance of cellular homeostasis under oxidative stress, establishing a vicious cycle that ultimately leads to the occurrence of OA. Figure made by Biorender.com.

Although Pathological changes of OA can affect all joint structures, degradation and loss of articular cartilage is a central feature (O’Neill and Felson, 2018), initially presenting as focal and heterogeneous forms of cartilage loss. Cartilage degradation in OA results from a disruption in homeostasis due to the activation of the chondrocytes by various factors that promote the production of matrix-degrading enzymes in excess of the capacity of the chondrocyte to replace damaged and degraded matrix components (Figure 1). Additionally, this process is also accompanied by subchondral plate thickening and hardening of the subchondral plate, osteophyte formation at the joint edges, joint capsule traction, mild synovitis of the affected joint, degeneration of the knee joint meniscus, and muscle weakness surrounding the joint.

As of now, there is no specific treatment for Osteoarthritis, and clinical management mainly relies on nonsteroidal anti-inflammatory drugs (NSAIDs) and tramadol to alleviate pain symptoms. However, these medications do not have the ability to delay or reverse the process of OA cartilage degeneration. Unfortunately, NSAIDs have gastrointestinal and cardiovascular side effects, making them unsuitable for long-term OA treatment. Consequently, there remains a lack of safe and effective drugs that can prevent or reverse the progression of OA (Chen et al., 2008). It is crucial to conduct in-depth research into the pathogenesis of OA to identify potential therapeutic targets.

In recent years, the role of autophagy in osteoarthritis (OA) has been increasingly recognized, and numerous studies have indicated that the dysregulation of chondrocyte autophagy is one of the main factors leading to cartilage damage. Autophagy, as a highly conserved degradation process, prevents cell damage and promotes survival by participating in various cellular processes such as epigenetic modifications, cell metabolism, cell senescence, and apoptosis in times of energy deficiency or exposure to external harmful stimuli (Chang, 2020). Studies have shown that chondrocyte autophagy plays a key role in maintaining cartilage homeostasis (Valenti et al., 2021). Upregulating chondrocyte autophagy levels in OA animal models cannot only reverse cartilage destruction but also inhibit inflammatory responses. In light of these findings, this review aims to explore the relationship between chondrocyte autophagy and OA, systematically discussing the complex interplay between autophagy and chondrocyte senescence, as well as the relevant regulatory molecules and signaling pathways, especially investigating the mechanisms by which non-coding RNAs and exosomes regulate chondrocyte autophagy. At the same time, potential therapeutic approaches targeting chondrocyte autophagy for OA were summarized. By doing so, we hope to provide references for clinical diagnosis and treatment as well as scientific research on osteoarthritis.

## 2 Chondrocytes play an important role in maintaining articular cartilage homeostasis

Articular cartilage consists of chondrocytes and their surrounding extracellular matrices (ECM). Chondrocytes represent 1%–5% of the total volume of Articular cartilage (Peng et al., 2021). Nevertheless, chondrocytes, being the sole cells of articular cartilage, synthesize ECM components and the majority of catabolic enzymes. They are considered to play an important role in maintaining Articular cartilage homeostasis (Fujii et al., 2022). Under physiological conditions, chondrocytes are in a state of low metabolic activity and maintain ECM components in a low-turnover state of equilibrium (Riegger and Brenner, 2020). This reduced metabolic activity could be attributed to a lack of blood supply in the cartilage, resulting in a relatively hypoxic and nutrient-deficient environment (Charlier et al., 2019). Cartilage homeostasis involves a balanced state of chondrocyte anabolism and catabolism. This equilibrium can be disrupted by various factors including excessive and abnormal mechanical loading, pro-inflammatory cytokines, oxidative stress, chemokines, and changes in growth factor responses and matrix during aging that contribute to structural changes in the surrounding ECM (Peng et al., 2021). The initial disruption of cartilage homeostasis manifests as organelle dysfunction in chondrocytes. This includes the generation of ROS, promoting the expression of matrix-degrading enzymes such as disintegrin and metalloproteinases with thrombospondin motifs 4 (ADAMTS-4) and ADAMTS-5, along with matrix metalloproteinase 13 (MMP13), Furthermore, this dysfunction reduces the ability of chondrocytes to synthesize ECM components, ultimately leading to the loss of cartilage matrix (Bolduc et al., 2019). The loss of cartilage matrix prevents the normal transmission of mechanical stress to chondrocytes, further disrupting intracellular homeostasis. Notably, dysfunctional autophagy and apoptosis in aged chondrocytes contribute to impaired maintenance of cellular homeostasis under oxidative stress, establishing a vicious cycle that ultimately leads to the occurrence of OA (Dikic and Elazar, 2018).

## 3 Autophagy

### 3.1 Definition and classification of autophagy

**FIGURE 2:**
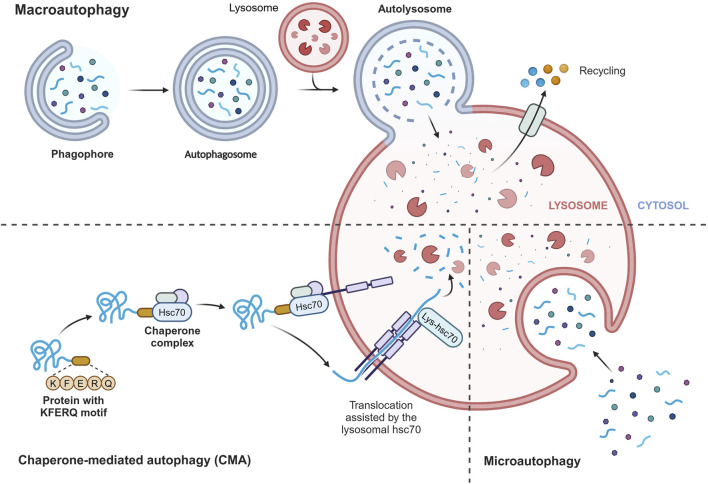
Three main types of autophagy. Chaperone-mediated autophagy (CMA) directly transports proteins to the lysosomal membrane, recognizing the KFERQ motif by heat shock 70 kDa protein 8 (HSPA8/HSC70), without involving the formation of a membrane. Microautophagy involves the invagination of the lysosome membrane to directly ingest cargo, while macroautophagy entails the formation of a double membrane. Despite their differences in transport mechanisms, all three types of autophagy eventually deliver cargo to the lysosome for degradation and recycling. Figure made by Biorender.com.

Autophagy is a highly conserved catabolic process in all eukaryotes, functioning as an intracellular scavenger to maintain intracellular balance (Dikic and Elazar, 2018). In mammalian cells, there are three main types of autophagy: chaperone-mediated autophagy (CMA), microautophagy, and macroautophagy (Parzych and Klionsky, 2014) (Figure 2). Despite their differences in transport mechanisms, all three types of autophagy eventually deliver cargo to the lysosome for degradation and recycling. CMA directly transports proteins to the lysosome membrane without involving the formation of a membrane. Microautophagy involves the invagination of the lysosome membrane to directly ingest cargo, while macroautophagy entails the formation of a double membrane. The specific process of microautophagy is shown below. Among these types, macroautophagy is the most extensively studied, and in this review, we will refer to it simply as autophagy. Autophagy primarily serves a cytoprotective function and requires tight regulation to effectively respond to the different cellular stimuli, enabling cells to adapt to changing environments.

### 3.2 Processes and regulation of autophagy

**FIGURE 3:**
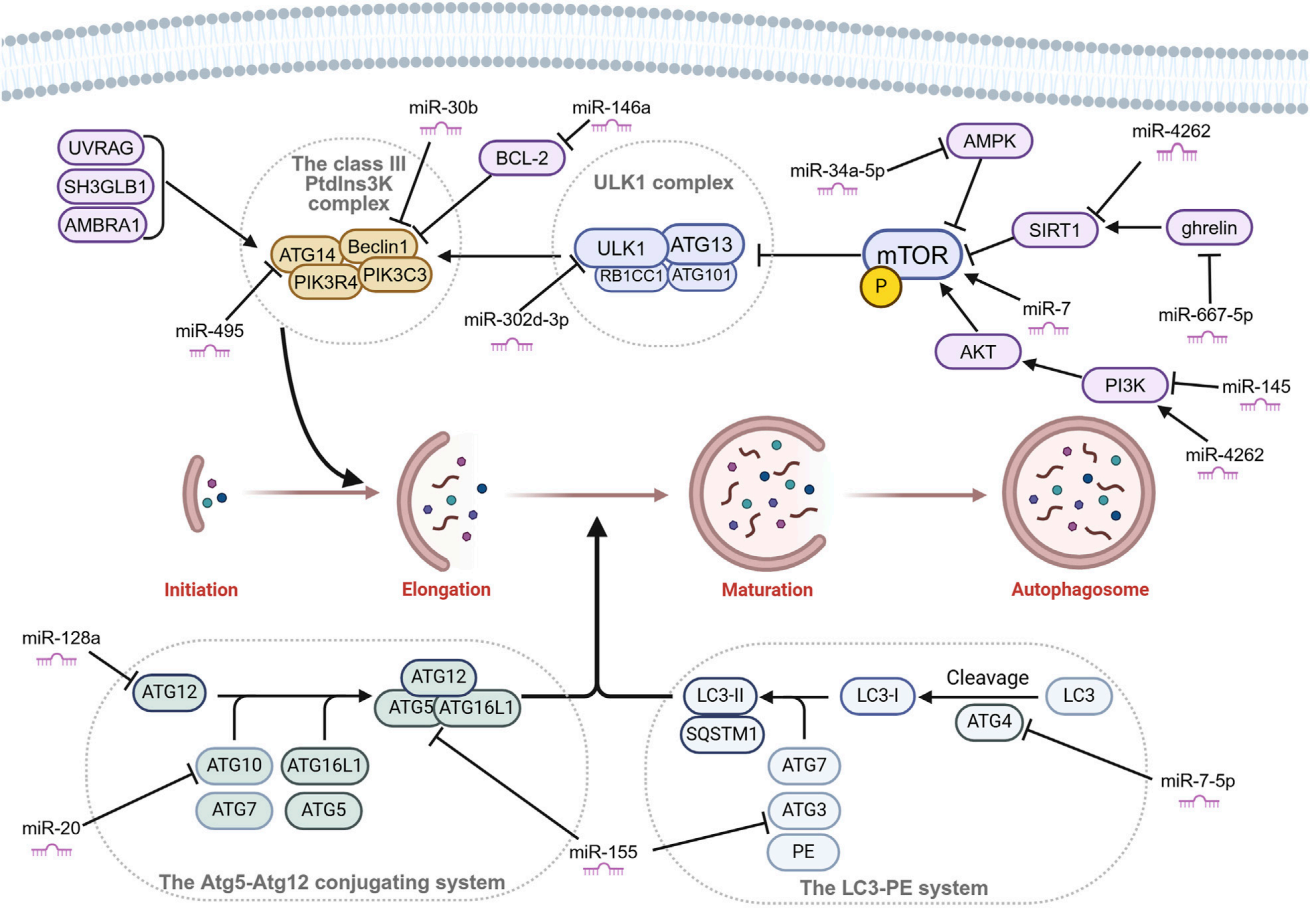
Autophagosome formation and miRNAs that affect chondrocyte autophagy by targeting key molecules in OA. Autophagosome formation is divided into three sequential stages: initiation, nucleation, and elongation. Many microRNAs (miRNAs) affect the key molecules of autophagy in OA. Figure made by Biorender.com.

The entire mechanism of autophagy involves a series of crucial steps: autophagosome formation, selection of cargo, autophagolysosome formation, and degradation (Ravanan et al., 2017). Each of these steps is essential for the autophagy process to take place successfully. Autophagy is initiated by autophagosome formation, which is divided into three sequential stages: initiation, nucleation, and elongation. The first step is the initiation, which mainly involves the Unc-51-like kinase 1 (ULK1) complex. The ULK1 complex is made up of ULK1 kinase, autophagy-related gene (ATG) 13, RB1CC1/FIP200 and C12orf44/ATG101. Under nutrient-rich conditions, mechanistic target of rapamycin complex 1 (MTORC1) binds to the complex and inactivates ULK1 and ATG13 by phosphorylation. However, during starvation, MTORC1 separates from the complex, and ATG13 and ULK1 become partially dephosphorylated. As a result, the complex is activated, initiating autophagy. RB1CC1/FIP200 and C12orf44/ATG101 are also associated with the induction complex and are essential for autophagy. RB1CC1/FIP200 may be the ortholog of yeast Atg17. Recent studies have indicated that Atg101, previously considered an accessory subunit, plays a significant role as a signaling hub within the ULK1 complex (Kim et al., 2018). ATG101 within the ULK1 complex plays a key role as a signaling hub, such as stabilizing ATG13 by heterodimer formation, the interaction with the PtdIns3K complex via the C-terminal region, and the interaction with other downstream factors via the WF finger. The second step is the nucleation, which primarily involves the class III PtdIns3K complex madding up of ATG14, Beclin1, PIK3C3, and PIK3R4. The class III PtdIns3K complex can be positively regulated by UV radiation resistance-associated (UVRAG), SH3 domain containing GRB2 like endophilin B1 (SH3GLB1), and autophagy and Beclin1 regulator 1 (AMBRA1). As well as, it can be negatively regulated by Bcl-2 binding to Beclin1 and preventing association with the complex (Boya et al., 2013). Subsequently, the elongation step is driven by 2 ubiquitin-like conjugation systems: the Atg5-Atg12 conjugation system and the LC3-PE system (Ohsumi and Mizushima, 2004). The Atg5-Atg12 conjugation system involves other members such as Atg7 (E1-like enzyme), Atg10 (E2-like enzyme), and Atg16L1 and eventually forms a trimeric or multimeric complex Atg5-Atg12-Atg16L1. This complex has been shown to possess an E3 ubiquitin ligase-like activity, promoting the transfer of Atg8/LC3 from Atg3 to membrane-localized PE (Kuang et al., 2013) (Figure 3). When the LC3-PE system is formed, this elongates and seals the phagophore to form a double membrane structure called the autophagosome. The autophagosomes then fuse to the lysosomes to form autophagolysosomes. The cargo present within the autophagosome is then transported to the lysosomes where they get degraded by the lysosomal enzymes (Geng and Klionsky, 2008).

## 4 Autophagy in articular cartilage of osteoarthritis

### 4.1 The role of autophagy in OA

Autophagy is an intracellular catabolic mechanism that plays a crucial role in removing dysfunctional organelles and macromolecules, thereby protecting cells against cellular stresses (Dikic and Elazar, 2018). The levels of Autophagy vary throughout the different stages of OA (Musumeci et al., 2015). During the early stages of OA, autophagy is activated in the superficial zones of the cartilage, possibly in response to cellular stress, to avoid cell death. However, in the late stages of OA, the expression of autophagy markers was significantly decreased in OA cartilage and chondrocytes, including ULK1, Beclin1, and LC3. Autophagy is vital for maintaining cellular homeostasis in normal adult articular cartilage; Several experiments have shown that an imbalance in chondrocyte autophagy is one of the key factors contributing to cartilage degradation in OA. Firstly, damage to autophagy reduces the ability of cartilage to overcome stressor challenges and nutrient deficiencies, making it difficult to maintain intracellular homeostasis, leading to an increase in the chondrocyte mortality rate and corresponding loss of the cartilage matrix. Secondly, the reduction of autophagy regulatory molecules can also lead to an increase in apoptosis in mouse models of OA. Thirdly, autophagy damage upregulates catabolism, such as increasing the production of degradation enzymes such as MMP13 and ADAMTS-5 in OA of human and animal models (Shapiro et al., 2014). Autophagy of chondrocytes not only participates in cellular metabolism, but also is involved in cell inflammation, apoptosis, and senescence, interacting with each other to affect OA. A study found that in a multicellular OA-environment culture model, the pro-inflammatory cytokine TNF-α decreased autophagy levels in chondrocytes. Activation of autophagy and downregulation of the inflammatory transcription factor NF-κB expression levels were observed after the addition of Calebin A. Reversible therapeutic effects were observed when the autophagy inhibitor 3-MA was added (Brockmueller et al., 2024). In Section 4.2, the interaction of autophagy with cell apoptosis and cell senescence is discussed in detail.

### 4.2 Effects of autophagy on chondrocyte apoptosis and chondrocyte senescence in OA

As age is the main factor in the pathogenesis of OA, a large number of studies have shown that a large number of aging-related changes have been found at the cellular level. Cellular senescence is characterized by a permanent proliferation arrest, triggered by a stress response to endogenous or exogenous damage, including telomere dysfunction, oncogene activation, and persistent DNA damage (Di Micco et al., 2021). Another hallmark of cellular senescence is the release of harmful pro-inflammatory molecules into the surrounding microenvironment, known as the senescence-associated secretory phenotype (SASP) (Coryell et al., 2021). The abnormal accumulation of senescent cells leads to chronic inflammation through SASP, promoting the development of OA. Strategies to limit the detrimental effects of senescence, such as eliminating senescent cells or regulating SASP, known as senotherapy, have emerged as potential treatments for aging-related conditions (Childs et al., 2017). In anterior cruciate ligment transection (ACLT) mice, selective elimination of senescent cells that accumulate in articular cartilage and the synovial membrane has shown promising results in attenuating the development of post-traumatic OA, relieving pain and promoting cartilage development (Jeon et al., 2017). There is a complex relationship between autophagy and cellular senescence. Initially, autophagy is thought to be an anti-aging mechanism with lifespan-prolonging effects (Mizushima and Komatsu, 2011). For example, Rapamycin, a mTOR inhibitor, downregulates the SASP and extends the lifespan of mice (Herranz et al., 2015). Autophagy can decelerate osteoarthritis progression by regulating cellular nutrient metabolism, and inflammation levels, and eliminating damaged organelles, thus postponing cellular aging and death. A recent study has found that IL-1β, an inflammatory factor commonly used to induce osteoarthritis *in vitro* models, inhibits chondrocyte autophagy, damaged autophagy leads to mitochondrial dysfunction and excessive production of ROS, ultimately resulting in senescence of chondrocytes (Xu et al., 2023). In addition, Recent research further confirms that methyltransferase-like 3 (METTL3)-mediated N6-methyladenosine (m6A) modification of ATG7 negatively regulated autophagy by attenuating RNA stability of ATG7, and impaired autophagy accelerated cellular senescence in a GATA4-dependent manner in OA-fibroblast-like synoviocytes (FLS) (Chen et al., 2022). Among them, GATA4 is a key regulator of senescence. Notably, autophagy can have a dichotomous effect on senescence. Current research on autophagy promoting aging mainly focuses on the post-transcriptional level in the field of OA. Lee et al. (2021) prove that Senescent chondrocytes increase and the abundance of KEAP1 (Kelch-like ECH-associated protein 1), eIF3 (eukaryotic initiation factor 3) components (eIF3B and eIF3L), and TNIP1 (TNFAIP3 interacting protein 1) protein decreases in the damaged region of human OA cartilage compared with that in the undamaged region through regulating of autophagy (Narita et al., 2011). p62-dependent selective autophagy degrades KEAP1 to activate the NRF2 pathway (Zhu et al., 2022), which promotes senescent cell survival via the attenuation of oxidative stress. Nuclear dot protein 52 kDa (NDP52)-dependent selective autophagy of eIF3 components facilitate senescent cells to secrete SASP. The function of TNIP1 expression varies in different conditions. In senescent cells, optineurin (OPTN)-dependent selective autophagy of TNIP1 destroys the negative feedback loop of the NF-KB pathway to promote the secretion of SASP, whereas TNIP1 expression increases SA-β-Gal activity in normal cells (Lee et al., 2021). The relationship between autophagy and senescence also remains inconclusive in many diseases, not just in the field of OA. The research shows that overexpression of one of the autophagy-related genes, ULK3, induces autophagy and senescence during oncogene-induced senescence (OIS) in IMR90 human diploid fibroblasts (HDFs). And knocking down the expression of autophagy-related genes caused SA-β-gal activity to be delayed, indicating that autophagy contributes to the establishment of senescence in HDFs (Lee et al., 2021). However, in contrast to this positive relationship, a study shows that selective autophagy degrades GATA4, which prevents senescence under normal conditions. So, upon senescence induction, GATA4 escapes from autophagic regulation and accumulative GATA4 promotes senescence via activating NFKB/NF-kB and the SASP, Whereas the mechanism of which is still unclear (Kang and Elledge, 2016). Therefore, autophagy inhibition may lead to different outcomes depending on the timing, duration, or type of its inhibition. However, In the field of OA, the molecular mechanism of autophagy activation to promote the establishment of senescent cells is almost absent, which may be a new target of treatment and deserves further study. Further study of its relationship may help the precisely regulating autophagy in the treatment of OA.

Indeed, multiple studies have demonstrated that apoptosis occurs more frequently in OA cartilage compared to normal cartilage (Charlier et al., 2016), especially in the late stages of OA (Zamli et al., 2013). In general, autophagy promotes cell survival, while apoptosis is a programmed cell death mechanism. The dysregulated balance between autophagy and apoptosis may be involved in the occurrence of OA (Sorice, 2022). Numerous investigations have revealed that in OA patients and animal models, chondrocyte apoptosis is increased, while autophagy levels are decreased (Zhang et al., 2021a). A study also found that α7-nAChRs deficiency mediates the switch from autophagy to apoptosis in primary chondrocytes in OA and mTOR pathway might be a key link in the association between autophagy and apoptosis via α7-nAChR activation (Liu et al., 2021). The quest for medicines that can maintain chondrocyte stability by balancing cell autophagy and apoptosis holds promise.

## 5 Key molecules regulated the autophagy pathway in OA

### 5.1 Non-coding RNA

#### 5.1.1 Introduction of non-coding RNA

**FIGURE 4:**
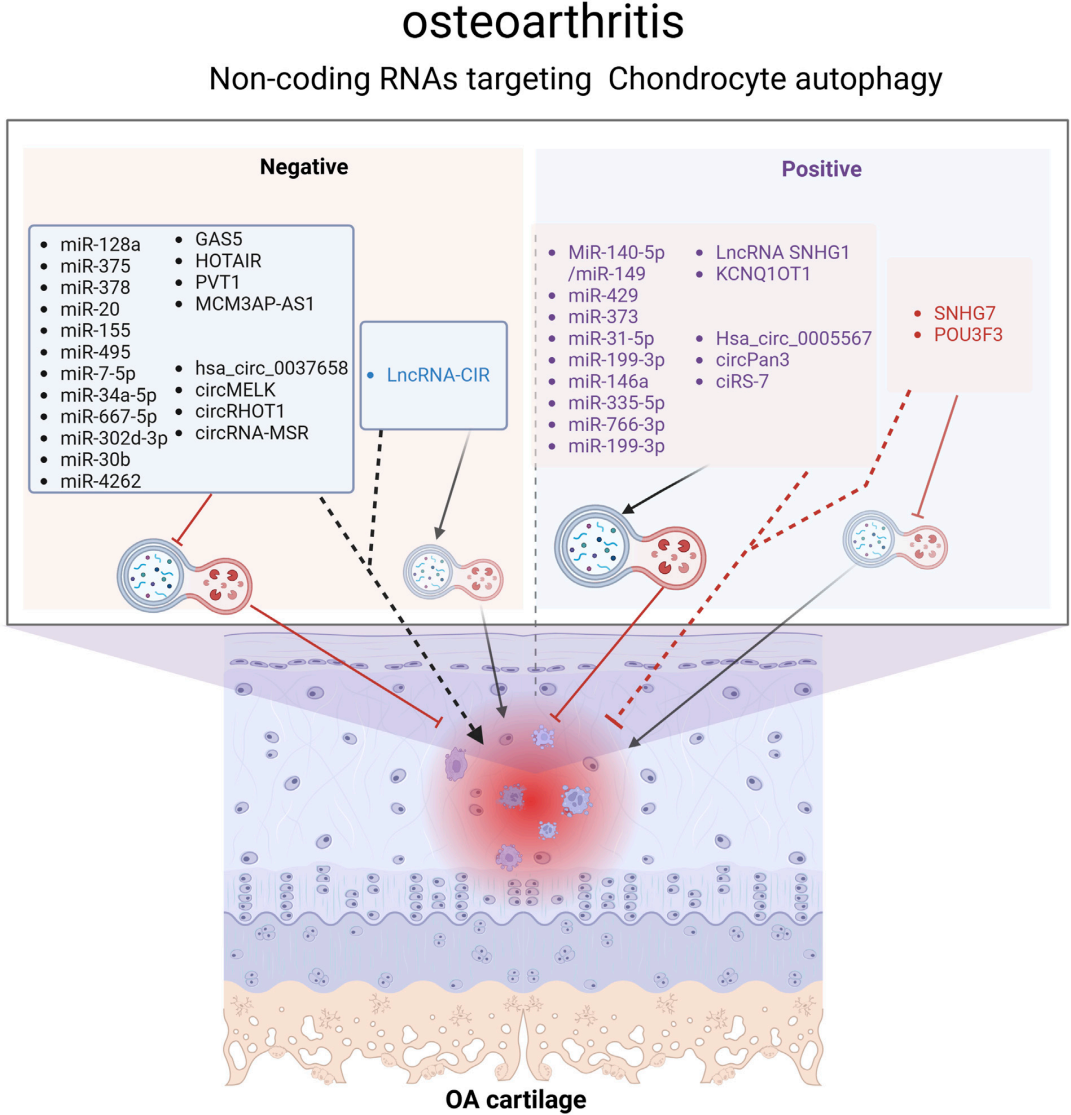
Non-coding RNAs (ncRNAs) regulating the chondrocyte autophagy pathway in OA. ncRNAs represented by black fonts aggravate OA by inhibiting chondrocyte autophagy, while those represented by blue fonts worsen OA by activating chondrocyte autophagy. ncRNAs represented by purple fonts alleviate OA by activating chondrocyte autophagy, whereas those represented by red fonts relieve OA by inhibiting chondrocyte autophagy. Overall, most studies demonstrate that chondrocyte autophagy is a protective mechanism for maintaining chondrocyte homeostasis. Figure made by Biorender.com.

It is true that 98% of the genome is transcribed, and the majority of these transcribed RNAs do not encode proteins, making them non-coding RNAs (ncRNAs) (Esteller, 2011). Despite not encoding proteins, ncRNAs are not devoid of function. In recent decades, research on ncRNAs have proved that they can influence the expression of other genes by participating in the regulation of transcriptional and post-transcriptional gene expression, contributing to the development of many diseases, including cancer (Anastasiadou et al., 2018), neurodegenerative diseases (Subhramanyam and Hu, 2017), autoimmune diseases, and cardiovascular diseases (Kleeberger et al., 2020). ncRNAs are divided into two categories: small noncoding RNAs and long noncoding RNAs. The most extensively studied ncRNAs to date include microRNA (miRNA), long non-coding RNA (lncRNA), and circular RNA (circRNA). There is substantial evidence suggesting that ncRNAs are involved in autophagy, and they can affect autophagy through different mechanisms, which is crucial in the pathogenesis of osteoarthritis (Ghafouri-Fard et al., 2022). As the understanding of the role of ncRNA in the regulation of autophagy, an increasing number of studies have found that ncRNA is involved in the regulation of autophagy in osteoarthritis (Figure 4).

#### 5.1.2 microRNA

MiRNAs are a conserved group of small RNAs consist of 20–22 nucleotides. They play a crucial role in mediating the post-transcriptional regulation of protein-coding genes by binding to the 3′-untranslated region (3′-UTR) of specifically targeted mRNAs, leading to mRNA degeneration and the suppression of their translation (Bartel, 2004). An increasing number of studies have confirmed that miRNA is involved in modulating the autophagy of OA chondrocytes (Table 1).

**TABLE 1:** The microRNA regulating chondrocyte autophagy in OA.

microRNA	microRNA expression	Affected tissue	Target/Signaling pathway	Results
miR-128a (Lian et al., 2018)	Upregulated	Human OA cartilage and cartilage of OA rat	Atg12	Autophagy ↓ Apoptosis ↑ ECM degradation↑
miR-375 (Li et al., 2020a)	Upregulated	Human OA cartilage, cartilage of OA mouse and miRNA-375 mimics transfected chondrocytes	ATG2B	Autophagy ↓ Endoplasmic reticulum stress ↑
miR-378 (Feng et al., 2022)	Upregulated	Human OA cartilage and cartilage of miR-378 transgenic mice	ATG2A; SOX6	Autophagy ↓ BMSC chondrogenic differentiation↓
miR-20 (He and Cheng, 2018)	Upregulated	Human OA cartilage and IL-1β induced OA chondrocyte	ATG10; PI3K/AKT/mTOR axis	Autophagy ↓ Cell proliferation ↓ Inflammation ↑
miR-155 (D’Adamo et al., 2016)	Upregulated	Human primary chondrocytes	Autophagy-related proteins	Autophagy ↓
miR-155 (Liu et al., 2022)	Upregulated	Polychlorinated biphenyls reduced OA chondrocyte	RICTOR/Akt/mTOR signaling	Autophagy ↓ ECM degradation↑
miR-302d-3p (Wang et al., 2019)	Upregulated	Human OA cartilage and miR-302d-3p mimics transfected chondrocytes	ULK1	Autophagy ↓ Cell proliferation ↓ Apoptosis ↑ Inflammation ↑
miR-30b (Chen et al., 2016)	Upregulated	TNF-α induced OA chondrocyte	BECN1 and ATG5	Autophagy ↓ Apoptosis ↑ ECM degradation↑
miR-7-5p (Zhao et al., 2023a)	Upregulated	Cartilage of OA rat and IL-1β induced OA chondrocyte	ATG4A	Autophagy ↓ ECM degradation↑
miR-34a-5p (Wang et al., 2024a)	Upregulated	Developmental dysplasia of the hip reduced OA	SESN2; AMPK/mTOR axis	Autophagy ↓ Cell proliferation ↓ cell migration ↓
miR-667-5p (Zeng et al., 2021)	Upregulated	Cartilage of OA rat and IL-1β induced OA chondrocyte	Ghrelin	Autophagy ↓ ECM degradation↑
miR-4262 (Sun et al., 2018)	Upregulated	TNF-α induced OA chondrocyte	SIRT1; PI3K/AKT/mTOR axis	Autophagy↓ Apoptosis↑ Cell viability↓
miR-140-5p/miR-149 (Wang et al., 2018a)	Downregulated	Human OA cartilage and IL-1β induced OA chondrocyte	FUT1	Autophagy ↓ Apoptosis ↑ Cell proliferation ↓
miR-373 (Zhang et al., 2021b)	Downregulated	Human OA cartilage and oxidative stress inducer tert-butyl hydroperoxide induced OA chondrocyte	miR-373/METTL3/TFEB axis	Autophagy ↓ Apoptosis ↑
miR-145 (Tian et al., 2024)	Downregulated	Human OA cartilage	PI3K/Akt/mTOR axis	Autophagy ↓ Cell viability↓
miR-199-3p (Yan et al., 2024)	Downregulated	cartilage of OA mouse and OA chondrocytes	TCF4	Autophagy ↓ Apoptosis ↑ Inflammation ↑
miR-146a (Chen et al., 2017)	Upregulated	Hypoxia-induced primary chondrocytes	Traf6/IRAK1/Bcl-2 axis	Autophagy ↑ ECM degradation ↓
miR-335-5p (Zhong et al., 2019)	Downregulated	Human OA cartilage	miR-31-5p/SOX4/ERK/mTORC1 axis	Autophagy ↓ Cell proliferation ↓ Apoptosis ↑ Inflammation ↑
miR-31-5p (Xu et al., 2022a)	Downregulated	Human OA cartilage and IL-1β induced OA chondrocyte	miR-31-5p/SOX4/ERK/mTORC1axis	Autophagy ↓ Cell proliferation ↓ Apoptosis ↑
miR-766-3p (Li et al., 2020b)	Downregulated	IL-1β induced OA chondrocyte	miR-766-3p/AIFM1 axis	Autophagy ↓ Apoptosis ↑ ECM degradation↑

Several miRNAs had been found to influence OA progression by modulating the autophagy-related gene (ATG). For instance, miR-128a hinders Atg12 expression to repress chondrocyte autophagy by targeting the 3′-UTR of Atg12 and exacerbates OA (Lian et al., 2018). MiR-375 inhibit chondrocyte autophagy to promote ERs (endoplasmic reticulum stress) by targeting ATG2B and exacerbates knee osteoarthritis (Li et al., 2020a). By decreasing ATG2A and SOX6 expression, miR-378 inhibits chondrocyte autophagy to aggravate OA (Feng et al., 2022). By targeting ATG10, miR-20 represses chondrocyte autophagy in OA (He and Cheng, 2018). D’Adamo et al. (2016) found that miR-155 targeted several autophagy-related genes, including ATG3, ATG5, ATG14, ULK1, FOXO3, MAP1LC3, GABARAPL1, and aggravated OA via inhibiting chondrocyte autophagy. Interestingly, a study shown that miR-155 directly regulates RICTOR in polychlorinated biphenyls reduced OA chondrocyte, thereby activating the Akt/mTOR signaling pathway, leading to autophagy suppression, promotion of ECM degradation (Liu et al., 2022). In addition, compared with the control group, the expression of miR-7-5p in cartilage tissue of OA rats was significantly upregulated, and autophagy was inhibited by suppressing the expression of ATG4A (Zhao et al., 2023a). A study found that miR-302d-3p is significantly upregulated in the cartilage tissue of OA patients, and *in vitro* experiments further confirmed that miR-302d-3p inhibits chondrocyte proliferation by downregulating ULK1 expression, promoting cell apoptosis and inflammation (Wang et al., 2019). Chen et al. (2016) discovered that increased expression of miR-30b in TNF-α-treated ADTC5 cells directly targets BECN1 and ATG5 to suppress autophagy, leading to enhanced cellular apoptosis and ECM degradation. Conversely, antimiR-30b is able to reverse these effects.

In addition to modulating the ATG, some miRNAs have also regulated autophagy through other mechanisms to involved in OA. For example, in developmental dysplasia of the hip reduced OA, miR-34a-5p was found to inhibit the expression of SESN2, thereby suppressing chondrocyte autophagy and exacerbating osteoarthritis (Wang et al., 2024a). By targeting Ghrelin, miR-667-5p suppressed chondrocyte autophagy and promoted OA progression (Zeng et al., 2021). In TNF-α induced primary chondrocytes, there is a notable increase in the expression of miR-4262. MiR-4262 activates the PI3K/AKT/mTOR signaling pathway by directly targeting SIRT1, resulting in the suppression of cellular autophagy and viability, promotion of cell apoptosis, and degradation of ECM (Sun et al., 2018).

Interestingly, there are miRNAs that activate autophagy in OA. For instance, in human OA cartilage, miR-140-5p/miR-149 is downregulated, and overexpression of MiR-140-5p/miR-149 has been shown *in vitro* cell experiments to reduce chondrocyte apoptosis and enhance autophagy by targeting fucosyltransferase1 (FUT1) (Wang et al., 2018a). In the cartilage tissues of OA patients and mouse OA model, both CREB and miR-373 are downregulated. Overexpression of CREB enhances the expression of miR-373, leading to the downregulation of METTL3, reducing the inhibition of METTL3 on TFEB to alleviate OA injury by restoring the activity of chondrocyte autophagy (Zhang et al., 2021b). In patients with osteoarthritis, the expression of miR-145 is downregulated in cartilage, and further research has found that overexpression of miR-145 activates chondrocyte autophagy by targeting FRS2 (Tian et al., 2024). Yan et al. (2024) found that miR-199-3p is downregulated and TCF4 is upregulated in the cartilage tissue of OA mice, and verified that upregulation of miR-199-3p expression can enhance autophagy and reduce inflammation in OA chondrocytes by targeting TCF4. Chen et al. (2017) found that miR-146a is crucial in autophagy and apoptosis in OA, thought to promote chondrocyte autophagic activity by targeting Bcl-2. A study revealed that expression of miR-335-5p is decreased in OA chondrocytes. By re-expressing miR-335-5p, it is able to promote the survival of OA chondrocytes and reduce the expression of inflammatory factors through the activation of autophagy and inhibiting apoptosis (Zhong et al., 2019), but the specific mechanism is still unclear. MiR-31-5p is downregulated in OA cartilage tissues and IL-1β induced OA chondrocyte, and it promotes cell autophagy and proliferation by negatively regulating SOX4 to inhibit the activation of mTORC1 (Xu et al., 2022a). Compared with normal chondrocytes, the expression of miR-766-3p in OA chondrocytes is significantly decreased. Overexpression of miR-766-3p activates autophagy through AIFM1 suppression, leading to decreased chondrocyte apoptosis and ECM degradation, ultimately slowing down the progression of OA (Li et al., 2020b).

Although many miRNAs have been identified as related to autophagy of OA cartilage, the core mechanism of miRNA affecting the autophagy control network still needs to be further explored. So far, miRNA has become a significant regulatory molecule of OA and holds promise as a new target for treating OA.

#### 5.1.3 LncRNA

Long non-coding RNAs (lncRNAs) are RNA transcripts longer than 200 nucleotides that do not encode proteins. They are predominantly produced by RNA polymerases II and have the ability to regulate protein-coding genes both positive and negative (Wang and Chang, 2011). LncRNAs are newly discovered functional ncRNAs that play a wide regulatory role through diverse mechanisms in many diseases. As more and more research is done on lncRNA, Some LncRNAs have been shown to involve in OA by regulating autophagy. A significant number of the lncRNAs acted as competing endogenous RNAs (ceRNAs) to regulate autophagy in OA. For instance, GAS5 upregulation stimulates apoptosis and suppressed autophagy by targeting miR-21, thereby increasing cartilage degradation in OA (Song et al., 2014). Another study found that miR-144 is also a miRNA site absorbed by lncRNA GAS5 sponge (Ji et al., 2021). HOTAIR overexpression led to reduce autophagy and induce apoptosis by inhibiting MIR-130-3P in OA cartilage (He and Jiang, 2020). Therefore, regulation of these genes might be a potential mechanism that can be targeted by gene therapy of KOA. Other lncRNAs regulating chondrocyte autophagy in OA are listed in Table 2.

**TABLE 2:** The lncRNAs regulating chondrocyte autophagy in OA.

LncRNA	Disease expression	Affected tissue	Target/Signaling pathway	Results
GAS5 (Song et al., 2014)	Upregulated	Human OA cartilage, human OA chondrocytes and cartilage of OA mice	miR-21	Autophagy↓ Apoptosis↑ ECM degradation↑
GAS5 (Ji et al., 2021)	Upregulated	cartilage of OA rat	miR-144/mTOR	Autophagy↓ Apoptosis↑
HOTAIR (He and Jiang, 2020)	Upregulated	Human OA cartilage and pcDNA3.0-HOTAIR transfected chondrocytes	miR-130a-3p	Autophagy↓ Apoptosis↑ Cell viability↓
LncRNA-CIR (Wang et al., 2018b)	Upregulated	Human OA cartilage, IL-1β induced OA chondrocyte and cartilage of OA rat	Autophagy-related regulatory pathways (not yet clearly defined)	Autophagy↑ Apoptosis↑ ECM degradation↑
PVT1 (Lu et al., 2020)	Upregulated	Human OA cartilage and IL-1β induced chondrocytes	miR-27b-3p/TRAF3 axis	Autophagy ↓ Cell viability ↓ Apoptosis ↑ Inflammation ↑
SNHG7 (Tian et al., 2020)	Downregulated	Human OA cartilage and IL-1β induced OA chondrocyte	SNHG7/miR-34a-5p/SYVN1 axis	Autophagy ↑ Cell proliferation ↓ Apoptosis ↑
MCM3AP-AS1 (Xu et al., 2022b)	Upregulated	Human OA cartilage, cartilage of OA rats and IL-1β- induced C28/I2 cells	MCM3AP-AS1/miR-149-5p/Notch1 axis	Autophagy ↓ apoptosis ↑ ECM degradation ↑
POU3F3 (Shi et al., 2022)	Downregulated	Human OA cartilage, cartilage of OA mouse and IL-1β induced chondrocytes	POU3F3/miR-29a-3p/FOXO3 axis	Autophagy↑ cell viability ↓ apoptosis ↑ inflammatory↑
LncRNA SNHG1 (Wang et al., 2024b)	Downregulated	OA animal cartilage tissues and IL-1β induced chondrocytes	PI3K/Akt pathway	Autophagy↓ Apoptosis↑
KCNQ1OT1 (Sun et al., 2022)	Downregulated	OA animal cartilage tissues and PA-induced ATDC5 cells	KCNQ1OT1/miR-128-3p/SIRT1 axis	Autophagy↓ apoptosis ↑ inflammatory↑

#### 5.1.4 circRNA

Circular RNA (circRNA) is formed through a back-splicing process of precursor mRNA (pre-mRNA) in higher eukaryotes, resulting in a complete loop structure. Unlike linear RNA, circRNA lacks 5′cap and 3′poly(A) tails, making it resistant to digestion by RNases and conferring greater stability and conservation (Wang et al., 2022). In recent years, more and more reports have found that circRNAs play an important role in the biological function of ceRNA networks. CircRNAs can act as competitiveendogenous RNAs, competing with miRNAs to influence the stability of target RNAs or their translation, thereby regulating gene expression at the transcriptional level. For example, Hsa_circ_0005567 overexpression activated chondrocyte autophagy, reducing chondrocyte apoptosis by downregulating miR-495 expression. The chondroprotective effect of Hsa_circ_0005567 overexpression in IL-1β induced OA chondrocyte can be reversed by the autophagy inhibitor 3-MA (Zhang et al., 2020). In another study, Jing Zeng et al. found that CircPan3 overexpression promotes chondrocyte autophagy to protect against OA injury via sponging miR-667-5p in OA rat cartilage tissues (Zeng et al., 2021). Other circRNAs regulating chondrocyte autophagy in OA are listed in Table 3.

**TABLE 3:** The circRNAs regulating chondrocyte autophagy in OA.

circRNA	Disease expression	Affected tissue	Target/Signaling pathway	Results
Hsa_circ_0005567 (Zhang et al., 2020)	Downregulated	IL-1β induced OA chondrocyte	miR-495/ATG14	Autophagy↓ Apoptosis↑
circPan3 (Zeng et al., 2021)	Downregulated	Cartilage of OA rat and IL-1β induced OA chondrocyte	circPan3/miR-667-5p/ghrelin axis	Autophagy↓ ECM degradation ↑
ciRS-7 (Zhou et al., 2020)	Downregulated	IL-1β induced OA chondrocyte adding ciRS-7-siRNA	ciRS-7/miR-7; PI3K/AKT/mTOR axis	Autophagy↓ ECM degradation ↑
hsa_circ_0037658 (Sui et al., 2021)	Upregulated	IL-1β induced OA chondrocyte	LC3, ATG5, BECN1, p62, AIF, caspase3; collagen Ⅲ, MMP13, aggrecan	Autophagy↓ Cell proliferation ↓
circMELK (Zhang et al., 2022)	Upregulated	OA human cartilage and IL-1β induced OA chondrocyte	miR-497-5p/MYD88/NF-κB axis	Autophagy↓ Apoptosis↑
circRHOT1 (Man et al., 2022)	Upregulated	OA human cartilage and cartilage of OA rats	miR-142-5p/CCND1 Axis	Autophagy ↓ Apoptosis ↑ Chondrocyte proliferation↑
circFOXO3 (Zhao et al., 2022)	Downregulated	Cartilage of OA rat and IL-1β induced OA chondrocyte	FOXO3; PI3K/AKT pathway	Autophagy ↓ Apoptosis ↑ ECM degradation ↑
circRNA-MSR (Jia et al., 2022)	Upregulated	OA human cartilage and LPS induced OA chondrocyte	miR-761	Autophagy ↓ cells viability ↓

### 5.2 Exosomes regulating chondrocyte autophagy in OA

Exosomes are one of the categories of extracellular vesicles, formed by the fusion of the plasma membrane and multivesicular endosome, with a diameter of less than 150 nm. They can transfer various molecules from parent cells to recipient cells, including proteins, lipids, metabolites, mRNA, and regulatory miRNAs, promoting intercellular interactions (Rahnama et al., 2024).

In recent years, the role and therapeutic potential of exosomes in various diseases, including osteoarthritis, have been receiving increasing attention. A study detected statistically significant differences in the expression levels of 52 mRNAs and 294 ncRNAs in synovial exosomes of OA and control patients. Subsequent bioinformatic analysis revealed that 101 ncRNAs are enriched in the PI3K/Akt and autophagy pathways, suggesting that exosomes may be a potential approach to target autophagy for alleviating OA (Wu et al., 2022). Ji et al. (2023) found that exosomes in the synovial fluid of the OA group exhibit a significantly higher abundance and smaller size compared to the injury group. Furthermore, qRT-PCR analysis confirmed a significant downregulation of miR-182-5p in the synovial fluid of the OA group. Subsequent *in vitro* experiments revealed that miR-182-5p activates chondrocyte autophagy and reduces cell apoptosis by targeting the downregulation of TNFAIP8 expression, thus alleviating OA (Ji et al., 2023). Exosomes derived from vascular endothelial cells can be efficiently taken up by IL-1β induced OA chondrocytes, exacerbating osteoarthritis by inhibiting autophagy and inducing cell apoptosis (Yang et al., 2021).

More intriguingly, stem cell-derived exosomes are widely studied as a potential therapeutic approach (Janockova et al., 2021). The level of miR-429 in adipose tissue-derived stromal cell (ADSC) exosomes is higher than that in chondrocyte exosomes, and studies have shown that ADCS exosomes can promote chondrocyte autophagy by targeting FEZ2 to improve cartilage injury (Meng et al., 2023). Infrapatellar fat pad (IPFP) mesenchymal stem cells (MSCs) -derived exosomes (MSC^IPFP^-Exos) protect articular cartilage from damage and improve gait abnormalities in DMM-induced mouse OA models by maintaining cartilage homeostasis, likely through the inhibition of the mTOR signaling pathway activation of chondrocyte autophagy by miR100-5p (Wu et al., 2019). Both MSCs-Exo and fucoidan-pretreated MSCs-Exo (F-MSCs-Exo) can activate autophagy of OA chondrocytes, upregulate the synthesis of Collagen II and Aggrecan, and downregulate the levels of MMP-13 and ADAMTS4. F-MSCs-Exo demonstrate superior therapeutic effects, possibly attributed to the enrichment of miR-146b-5p in F-MSCs-Exo. miR-146b-5p alleviates OA-related pathological changes by targeting TRAF6 to inhibit the PI3K/AKT/mTOR pathway (Lou et al., 2023).

In addition, it is worth noting that exosomes have the advantages of biocompatibility, biodegradability, low toxicity, specificity to target cells, promoting membrane fusion, longer half-life, and no immune response, and are recognized as a new type of nanoscale delivery system (Rajput et al., 2022). Injecting Synovial mesenchymal stem cell-derived exosomes (SMSC-Exo) into the joint cavity of DMM-induced mouse OA models can improve OA-related pathological changes. The mechanism may be related to the higher expression of MATN3 in SMSC-Exo, which inhibits the PI3K/AKT/mTOR pathway to activate chondrocyte autophagy. Treatment of OA chondrocytes with oe-MATN3 lentivirus-treated SMSC-Exo yields more significant effects, indicating that SMSC-Exo is a drug carrier with therapeutic potential (Long et al., 2023).

### 5.3 Other key molecules regulating chondrocyte autophagy in OA

Indeed, the involvement of various molecules in autophagy regulation within OA provides valuable insights into the disease’s pathogenesis and potential therapeutic approaches. Tribbles homolog 3 (TRB3) protein levels have been found to be elevated in chondrocytes of OA patients. Gu et al. (2022) demonstrated that TRB3 knockdown activates chondrocytes autophagy by interacting with p62, thereby inhibiting chondrocyte senescence, as evidenced by reduced levels of p21 and p16. SIRT1 activation has been shown to enhance autophagy and alleviates OA cartilage degeneration. The mechanism of SIRT1’s action involves direct deacetylation of lysine residues on key autophagy proteins (Beclin1, ATG5, ATG7, LC3), leading to increased autophagy in chondrocytes, independent of mTOR/ULK1 signaling (Sacitharan et al., 2020). Moreover, SIRT1 can alleviate the severity of OA by down-regulating TEN and inhibiting the ubiquitination of EGFR to prevent ECM degradation and activate the autophagy of chondrocytes (Lu et al., 2022). SIRT3 inhibits PI3K/Akt/mTOR signal transduction, its Overexpression can protect cartilage and alleviate the severity of OA (Xu et al., 2021).

Forkhead box class O1 (FOXO1) is a Key downstream effector of the TGF-β/TAK1 non-standard signal pathway regulating articular cartilage homeostasis (Wang et al., 2020). The cartilage protective effect of FoxO1 on injury-induced OA indicates that targeted activation of FOXO1 is a novel and attractive candidate therapy for human OA. Recently, a research report said that Panobinostat, a histone deacetylase inhibitor (HDACI), targets FOXO1 to activate autophagy, meanwhile, reducing basal or IL-1β Induced inflammatory mediators and the ability of ECM to degrade the expression of enzymes in the knee of OA patients and destabilization of the medial meniscus (DMM)–induced mice OA models, but the side effects of systemic medication are large, especially in the blood system, and intra-articular administration may be the solution (Ohzono et al., 2023).

FBXO21, a subunit of the Skp1-cullin-F-box (SCF) ubiquitin E3 ligases, has been found to induce chondrocytes to release various enzymes (MMP3, MMP13, and ADAMTS5) by binding to ERK, leading ECM degradation. Additionally, FBXO21 inhibits autophagy and aggravates cartilage degeneration by phosphorylating ERK in MIA‐treated rats and IL‐1β‐treated rat chondrocytes, and knockdown FBXO21 significantly increases autophagy levels and reduces apoptosis, thus reducing ECM degradation (Lin et al., 2021).

These studies provide valuable insights into the role of autophagy in OA pathogenesis, and offer promising avenues for the development of novel therapeutic approaches for the treatment of osteoarthritis.

## 6 Prospective of treatment via regulating chondrocyte autophagy in OA

At present, the treatment of osteoarthritis includes medicine and non-drug treatment, but both are symptomatic treatments. In recent years, many researchers have deeply explored the autophagy mechanism of OA and recognized that autophagy damage is an important mechanism for the progress of osteoarthritis. Targeted autophagy regulation may hold promise for future disease-modifying therapies for patients with OA. As more and more molecules are found to promote or alleviate the condition of osteoarthritis by regulating autophagy, these molecules have become potential targets for the treatment of OA. The potential therapeutic methods targeting autophagy of cartilage can be mainly divided into drug therapy and non-drug therapy. Non-drug therapy can be further divided into gene therapy, stem cell therapy, and exercise therapy (Table 4).

**TABLE 4:** Potential therapeutic approaches targeting chondrocyte autophagy for OA.

Type	Name	Vector	Model	Target	Method of administration
Drug therapy	Rapamycin (Bao et al., 2020)	Cationic liposome–incorporating HMs	ACLT and DMM-induced rat OA models	mTOR	Intra-Articular Injection
	Epigallocatechin 3-Gallate (Huang et al., 2020)	Phosphate buffered saline (PBS)	ACLT-induced rat OA models	mTOR	Intra-Articular Injection
	Artemisin (Li et al., 2022)	Normal saline	DMM-induced rat OA models	TNFSF11; PI3K/AKT/mTOR axis	Intra-Articular Injection
	Four-octyl itaconate (Pan et al., 2022)	Normal saline	DMM-induced rat OA models	PI3K/AKT/mTOR axis	Oral administration
	Sodium butyrate (Zhou et al., 2021)	Normal saline	ACLT-induced mouse OA models	PI3K/AKT/mTOR axis	Oral administration
	Mulberroside A (Lu et al., 2023)	PEG300	DMM-induced mouse OA models	MAPK/NF-κB/PI3K-AKT-mTOR axis	Intra-Articular Injection
	Metformin (Xu et al., 2024a)	Normal saline	ACLT and DMM-induced rat OA models	PI3K/AKT/mTOR axis	Oral administration
	Shikimic acid (You et al., 2021)	Normal saline	ACLT-induced rat OA models	MAPK/NF-κB axis	Intra-Articular Injection
	Alantolactone (Pei et al., 2021)	20% SBE-β-CD + DMSO + Normal saline	DMM-induced mouse OA models	STAT3; NF-κB signal pathways	Intra-Articular Injection
	Inactivated *Lactobacillus* (Cho et al., 2022)	0.5% carboxymethylcellulose	MIA induced rat OA models	The gut environment and autophagic flux	Oral administration
Exercise therapy	Moderate-intensity exercise (Li et al., 2021)	NA	MIA induced rat OA models	P2X7/AMPK/mTOR axis	Treadmill exercise
Gene therapy	Negative ncRNAs (MiR-128a) (Lian et al., 2018)	Lentiviral vector	ACLT-induced rat OA models	Atg12	Intra-Articular Injection
	Positive ncRNAs (miR-199a-3p) (Zhao et al., 2023b)	MSCs^SC^-Exos	DMM-induced mouse OA models	mTOR	Intra-Articular Injection
Stem cell therapy	Allogeneic ADSCs	PBS	ACLT and DMM-induced rat OA models	miR-7-5p/ATG4A	Intra-Articular Injection
	An equal proportionate mixture of human ADSCs and SDSCs (Wu et al., 2024)	PBS	pMMx- induced nude rats OA models	FOXO1 signaling pathway	Intra-Articular Injection
	USCs (Xu et al., 2024b)	PBS	ACLT-induced rat OA models	Unclear	Intra-Articular Injection

MTOR is the most important autophagy-negative regulator. MTOR and its downstream signal transduction through the autophagy pathway may be a promising therapeutic strategy in OA (Zhang et al., 2015). Rapamycin (RAPA), a selective mTOR inhibitor, promote chondrocyte autophagy to alleviate OA (Bao et al., 2020). The water solubility of RAPA is poor and the concentration of drug injected into the joint cavity is low. Due to the shortcomings, the clinical application of RAPA in OA is limited. Many research found that Drug delivery systems (DDS) such as nanoparticles (NPs), microparticles, liposomes, or other vectors might overcome these limitations to enhance the efficacy of RAPA (Schulze-Tanzil, 2021). Lei et al. (2022) invented an injectable cationic liposome–incorporating HMs containing RAPA that can target articular cartilage and release RAPA to activate the autophagy of chondrocytes. Liposomes can also be used as a joint lubricant to reduce mechanical damage in OA. Huang et al. (2020) found that Epigallocatechin 3-Gallate (EGCG) reduced the expression of mTOR to enhance autophagy and reduce apoptosis. Injecting EGCG into the articular cavity could inhibit cartilage degeneration and inflammation in a rat model of OA established by ACLT (Huang et al., 2020). Experiments had proved that Sodium butyrate (NaB), artemisin (AT), Mulberroside A (MA), and Four-octyl itaconate (OI) promote autophagy of chondrocytes to alleviate OA by regulating P13K/AKT/mTOR signal pathway (Li et al., 2022; Pan et al., 2022; Zhou et al., 2021; Lu et al., 2023). The four drugs are administered in different ways. AT and MA are intra-articular injection, while NaB and OI are taken orally. Adenosine monophosphate-activated protein kinase (AMPK) is also a common therapeutic target for regulating autophagy (Saikia and Joseph, 2021). Na et al. (2021) found that metformin increased autophagy flux, inhibited the expression of inflammatory factors, and reduced cartilage damage in the MIA-induced rat OA models. In addition, the latest research found that metformin activates autophagy by inhibiting the PI3K/AKT/mTOR signaling pathway to improve cartilage injury in the cartilage of OA rats (Xu et al., 2024a). Cell and animal experiments had proved that Shikimic acid (SA), a natural ingredient extracted from Illicium verum, could delay the progress of OA by activating autophagy through AMPK/NF-kB pathway (You et al., 2021). Alantolactone (ALT), a sesquiterpene lactone compound, promoted autophagy of OA chondrocytes and improves cartilage degeneration by selectively inhibiting the expression of STAT3 in rat models of OA established by DMM (Pei et al., 2021). Cho et al. (2022) found that Inactivated *Lactobacillus* (LA-1) and butyrate regulated intestinal microecology and activated autophagy in MIA-induced rat OA models, meanwhile proposed that regulating intestinal microenvironment might become a future treatment strategy.

Besides drugs, moderate-intensity exercise also promoted chondrocyte autophagy to alleviate OA through the P2X7/AMPK/mTOR signal axis. In this research, moderate-intensity exercise refers to exercising 5 days a week for 1 h each day at a treadmill speed of 18 m per minute in monosodium iodoacetate (MIA)-induced rat OA models, with an intervention period of 4 weeks (Li et al., 2021).

With the increasing attention to the role of non-coding RNA in OA, gene therapy is being widely researched in the field of OA as a promising strategy, and may become a new drug benefiting OA patients (Chen et al., 2023). Multiple studies have shown that intra-articular injection of therapeutic nucleic acids is an effective method for treating osteoarthritis. MiR-128a is a negative miRNA that inhibits chondrocyte autophagy and promotes the progression of osteoarthritis. Intra-articular injection of lentivirus expressing miR-128a antisense oligonucleotides (miR-128a-AS) can knock down the endogenous expression of miR-128a in cartilage tissue, promoting autophagy levels and relieving osteoarthritis (Lian et al., 2018). The choice of gene vectors has also been widely researched, with options such as lipofectamine 3000, generation 5 (G5) polyamidoamine (PAMAM) dendrimer, lentiviral vector and exosomes. While these gene vectors can prevent RNA degradation in the joint cavity, they currently lack precise targeting of chondrocytes. Further research is needed to ensure safety due to the differing effects of most microRNAs on chondrocytes and synovial cells, which is currently impeding the progress of clinical studies. Therefore, developing an efficient gene vector that precisely targets chondrocytes is crucial (Endisha et al., 2021). Research on regulating autophagy-rich microRNA exosomes as a means to enhance cartilage repair in osteoarthritis has received increasing attention. Delightfully, Zhao et al. (2023b) have developed a vector based on subcutaneous fat (SC) stromal cells derived exosomes (MSCs^SC^-Exos), which can accurately deliver miR-199a-3p to cartilage tissue for cartilage repair. The cartilage-specific peptide (CAP) motif has been shown to effectively deliver miRNA to chondrocytes in the knee joint cartilage. This helps to further conduct large-scale animal experiments and clinical trials on protective miRNA for OA (Zhao et al., 2023b).

Stem cell therapy has become increasingly popular in recent years due to its potential to differentiate into various types of cells and repair damaged tissues, including cartilage tissue. Intra-articular injection of allogeneic Adipose-derived stromal cells (ADSCs) in ACLT and DMM-induced rat OA models can prevent cartilage damage and reduce ECM degradation. Further research found that ADSCs treatment can reverse the decreased autophagy level in OA chondrocytes, which is caused by decreasing miR-7-5p and increasing ATG4A expression levels (Zhao et al., 2023a). Recent studies have shown that injecting a mixture of human ADSCs and synovium-derived stem cells (SDSCs) in equal proportions into the knee joint cavity of OA rats can alleviate cartilage degeneration, synovial inflammation, and osteophyte formation. Through RNA sequencing technology and *in vitro* experiments, an equal proportionate mixture of ADSCs and SDSCs activates autophagy via the FOXO1 signaling pathway to alleviate pathological changes associated with OA (Wu et al., 2024). The latest research has also found that ADSCs can enhance the autophagy of OA chondrocytes and decrease the expression of matrix degradation-related proteins and inflammatory factors. It is worth noting that urine-derived stem cells (USCs) have a similar effect and are more effective than ADSCs (Xu et al., 2024b). Stem cell therapy is expected to become a treatment strategy for OA, but this requires more animal model experiments and clinical trials to further validate its effectiveness and safety (Jo et al., 2017; Tabet et al., 2024).

Unfortunately, there are still some potential therapeutic drugs that have not been successfully developed. Liu et al. (2021) found that knocking down 7-nAChR inhibited autophagy by enhancing the phosphorylation of mTOR. The activation of 7-nAChR might become a potential way to treat OA, whereas the side-effect-free 7-nAChRs-specific agonists have not been invented.

Although activation of autophagy can delay the degradation of OA cartilage, autophagy activators should not be blindly used. Rockel et al. (2020) proved that compared with the PBS group, D-isomerTAT-beclin-1 injected into the knee joint of DMM-induced mouse OA models showed no significant difference in knee cartilage degeneration, but significantly induced synovial hyperplasia.

Overall, the regulation of autophagy presents a promising avenue for the development of disease-modifying therapies for OA. Further research and understanding of the autophagy control network in OA will be essential for identifying and refining potential targets for effective and safe treatments.

## 7 Conclusion

Osteoarthritis is a common disease in China and the incidence rate increases with age. However, the current treatment methods are very limited, and symptomatic treatment can only alleviate symptoms, but cannot delay or reverse the process of OA. In recent years, a large number of experimental studies have been carried out to explore the pathogenesis of OA and try to find an effective and safe drug. Autophagy, as a compensatory adaptation mechanism, is activated at the early stage of OA and is in a state of inhibition as the disease progresses. Although some molecularly targeted inhibition of autophagy can inhibit cartilage degradation, it is generally believed that activation of autophagy can improve OA. Experiments have found that many molecules can target chondrocytes to regulate autophagy to alleviate OA, which brings hope to OA patients. Since Senescent chondrocytes are a factor that aggravates the pathological changes of osteoarthritis, the dual effects of autophagy on senescence deserve attention and in-depth study. Autophagy, apoptosis, and aging have been paid attention to in the pathogenesis of OA, although these three interact in mechanism, autophagy is at the core of it. We believe that finding a balance between autophagy, apoptosis, and aging to maintain the homeostasis of chondrocytes may be a potential treatment for OA.
